# Cirrhosis and frailty assessment in elderly patients: A paradoxical result

**DOI:** 10.1097/MD.0000000000018501

**Published:** 2020-01-10

**Authors:** Alessandro Federico, Giuseppe Gerardo Caprio, Anna Maria Dalise, Michelangela Barbieri, Marcello Dallio, Carmelina Loguercio, Giuseppe Paolisso, Maria Rosaria Rizzo

**Affiliations:** a Department of Precision Medicine; b Department of Advanced Medical and Surgical Sciences - University of Campania Luigi Vanvitelli Naples, Italy.

**Keywords:** aging, cirrhosis, frailty

## Abstract

The frailty represents a key determinant of elderly clinical assessment, especially because it allows the identification of risk factors potentially modifiable by clinical and therapeutic interventions. The frailty assessment in elderly patients usually is made by using of Fried criteria. However, to assess the frailty in cirrhotic patients, multiple but different tools are used by researchers. Thus, we aimed to compare frailty prevalence in elderly patients with well-compensated liver cirrhosis and without cirrhosis, according to Fried criteria.

Among 205 elderly patients screened, a total of 148 patients were enrolled. The patients were divided into 2 groups according to the presence/absence of well-compensated liver cirrhosis.

After clinical examination with conventional scores of cirrhosis, all patients underwent anthropometric measurements, nutritional, biochemical, comorbidity, and cognitive performances. Frailty assessment was evaluated according to Fried frailty criteria.

Unexpectedly, according to the Fried criteria, non-cirrhotic patients were frailer (14.2%) than well-compensated liver cirrhotic patients (7.5%). The most represented Fried criterion was the unintentional weight loss in non-cirrhotic patients (10.1%) compared to well-compensated liver cirrhotic patients (1.4%). Moreover, cumulative illness rating scale -G severity score was significantly and positively associated with frailty status (*r* = 0.234, *P* < .004). In a multivariate linear regression model, only female gender, body mass index and mini nutritional assessment resulted associated with frailty status, independently of other confounding variables.

Despite the fact that elderly cirrhotic patients are considered to be frailer than the non-cirrhotic elderly patient, relying solely on “mere visual appearance,” our data show that paradoxically non-cirrhotic elderly patients are frailer than elderly well-compensated liver cirrhotic patients. Thus, clinical implication of this finding is that frailty assessment performed in the well-compensated liver cirrhotic patient can identify those cirrhotic patients who may benefit from tailored interventions similarly to non-cirrhotic elderly patients.

## 1 Introduction

Over the last years, frailty in elderly patients is considered the most powerful predictor of disability and other adverse events, including institutionalization and mortality.^[1]^ Frailty is characterized by increased vulnerability, resulting from age-associated declines in the ability of recovering after acute stressful events.^[2]^ Fried and colleagues defined frailty as a heterogeneous clinical syndrome characterized by the presence of 3 or more of 5 domains suggesting decreased physiologic reserve^[3]^ (Table 1). A pre-frail stage, identifying the patients at high risk of developing frailty, is characterized by presence of 1 or 2 criteria. Such criteria define a new concept different from disability or comorbidity. In fact, patients with comorbidity or disabilities might not be frail and frail patients might not be disabled. Therefore, the frailty represents a key determinant of elderly clinical assessment,^[4]^ especially because it allows the identification of risk factors potentially modifiable by clinical and therapeutic interventions. Although the frailty is a concept applied to elderly patients, several investigators have reported that frailty assessment predict mortality, length of hospitalization and rehabilitation for not elderly patients with cirrhosis.^[5–7]^ However, to assess the frailty in cirrhotic patients, multiple different tools are used by researchers. Dunn et al^[8]^ evaluated frailty using only 2 (gait speed and grip strength) of the 5 Fried parameters.^[3]^ Lai et al^[9]^ used only the measures related to physical performance because they believe that some parameters recommended by Fried et al^[3]^ are limited by the inclusion of self-reported components. Importantly, in cirrhotic patients Fried Frailty criteria were used mostly in the assessment of liver transplant as prognostic value of decompensated patients with higher levels of model for endstage liver disease (MELD).^[10,11]^ MELD score is used mainly to provide risk stratification prior to allocate the patients on waitlist for liver transplants. Other scores, as Child-Pugh score or Acute Physiology and Chronic Health Evaluation^[12]^ or chronic liver failure-sequential organ failure assessment scores,^[13]^ indicates whether the cirrhotic patients are well-compensated or decompensated, but neither of this included measures of multidimensional and/or frailty assessment that are instead typical for evaluating the elderly patient. Interestingly, recent studies show that the prevalence of chronic liver disease is increasing in the elderly population.^[14]^ Among European countries, the highest prevalence of hepatitis C virus (HCV) infection has been found in Italy. Such prevalence is ranging between 3% and 26%, and a progressive increase along with age has been observed, in particular in southern regions of Italy.^[15]^ This increase was mainly due to the aging HCV cohort and rise in fatty liver disease^[16]^; older patients are becoming the most representative part of those seen in outpatient clinic visits for HCV and epidemiological data suggest that they will even increase in the next future.^[17]^ This could be explained by the new-entry of several anti-HCV drugs, and by the possibility to combine them in safe and effective anti-viral regimens, with high grades of sustained viral response even in elderly.^[18,19]^

**Table 1:**
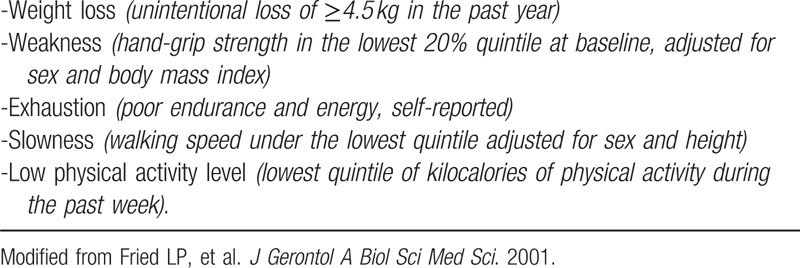
The frailty phenotype (FP): criteria and measurement.

Indeed, studies investigating the frailty assessment in elderly well-compensated liver cirrhotic compared to elderly non-cirrhotic, are thus far lacking. In this study we aimed to compare frailty prevalence in elderly patients with well-compensated liver cirrhosis and without cirrhosis, according to Fried criteria.

## 2 Methods

### 2.1 Study population

The study population consisted of elderly patients aged ≥65 years. affected and not affected by HCV-related cirrhosis, consecutively admitted from September 2015 to January 2018 at Hepato-gastroenterologic and at Geriatric Centre of our University Hospital. Two hundred five elderly patients were screened and 148 enrolled, according to the inclusion and exclusion study criteria, after signing informed consent. Inclusion criteria were: age ≥65 years, presence or absence of HCV-related cirrhosis CHILD A. Exclusion criteria were: age <65 years, recent acute illness, decompensated cirrhosis, severe cognitive decline and/or Alzheimer dementia, drugs or alcohol abuse or dependence in the last 2 years, drug therapy or life style modified within the 3 months before the study.

The definition of the presence/absence of HCV cirrhosis, the etiology and the staging of the disease were diagnosed after exclusion of other causes of liver diseases, by serological tests and clinical and instrumental data. All patients were enrolled before starting an antiviral therapy for HCV infection. After a clear explanation of the study, all patients provided written informed consent to participate in the study. The study was conduct in accordance with the Declaration of Helsinki and the protocol was approved by the Ethics Committee of our Institution/University Hospital (Project identification code: n 416/2015).

### 2.2 Clinical examination

Anthropometric, biochemical, and cognitive parameters were recorded. Baseline questionnaires were used to gather information on clinical evaluations including physical examination, vital signs, nutritional status, lifestyle within the 3 months before the study. Physical functioning was measured with the physical component summary (PCS) from the Short Form-12 Health Survey.^[20,21]^ The PCS asked if a patient's health limited his/her ability to perform moderate activities, to climb several flights of stairs, to accomplish daily activities, or to be involved in work or daily activities, and if pain interfered with normal activities. The PCS has a range of 0 to 100 with a mean score of 50 and a standard deviation (SD) of 10 in the general U.S. population. A higher score indicates better functioning (data not shown).

### 2.3 Laboratory measurements

An overnight fast of at least 12 hours preceded insertion of an antecubital vein catheter for blood collection. Clinical and standard baseline biochemical parameters were assessed, including liver function: complete blood count, fasting plasma glucose (FPG), cholesterol, triglycerides, glutamic oxalacetic transaminase, glutamic pyruvic transaminase, gamma -glutamyltransferase, bilirubin, albumin, creatinine, anti-HCV antibody, HCV-ribonucleic acid.

#### 2.3.1 Evaluation of cirrhosis with conventional scores

Child-Pugh score is used to evaluate the stage and the prognosis of chronic liver disease and cirrhosis. According to the sum of 5 clinical features, patients can be categorized into Child-Pugh grades A (5 to 6 points), B (7 to 9 points), or C (10 to 15 points).^[17,22]^

To exclude a possible alcohol-induced aetiology of cirrhosis, we used the alcohol use disorders identification test; it is an alcohol screen that identifies patients who are hazardous drinkers or have active alcohol use disorders. In men a score of 4 or more is considered positive; in women, a score of 3 or more is considered positive.^[18,23]^

#### 2.3.2 Frailty assessments

Assessments of physical frailty were performed in the outpatient setting using the Fried Frailty criteria,^[3]^ a mixed performance-based and self-report phenotype model of frailty. These criteria include 5 components:

(1) Unintentional weight loss of ≥4.5 kg in the last year (reported by patient; score 1 = yes weight loss, score 0 = no weight loss);
(2) Weakness (assessment obtained by handgrip strength measurement, by Kern Dynamometer.^[24]^ This measurement was performed 3 times, with a 1-minute rest interval between measurements, considering the highest values. Muscle weakness values <20 kg and <30 kg values, were considered for female and males, respectively, to indicate poor muscle strength.^[25]^ The interpretation of results takes into account sex and body mass index (BMI); score 1 = yes weakness; score 0 = no weakness);
(3) Exhaustion (score 1 or 2 = fatigue or exhaustion felt most of the time; score 0 = fatigue or exhaustion felt rarely or not at all).
(4) Slow gait (walking time over a distance of 4. m; interpretation of results takes into account sex and height)^[26]^;
(5) Low physical activity (physical activity weekly rate); score 1 = yes; score 0 = no.^[27]^

Frail was defined as Fried Frailty ≥3 points out of a maximum of 5 (Table 1).

#### 2.3.3 Multidimensional assessment evaluation

Global cognitive function was assessed with mini-mental state examination corrected for educational levels^[28]^ and with montreal cognitive assessment test (MoCA).^[29]^ Activity functions were assessed by instrumental activities of daily living (IADL) and the basic activities of daily living (BADL)^[30,31]^ while depressive symptoms by geriatric depression scale (GDS short version).^[32]^ The possible neuropsychiatric symptoms or behavioural alteration and the stress of the caregiver were evaluated by neuropsychiatric inventory test (NPI).^[33]^

Comorbidity was assessed with the cumulative illness rating scale geriatric version (CIRS-G).^[34]^ The score differentiates between 14 organ systems. Every comorbidity of a patient was assigned to one of the organ systems and rated from 1 (mild comorbidity) to 4 (extremely severe comorbidity). In general, the levels were defined as: level 0: no comorbidity; level 1: current mild problem or past significant problem; level 2: moderate disability or morbidity/requires “first line” therapy; level 3: severe or constant significant disability/“uncontrollable” chronic problems; level 4: extremely severe/immediate treatment required/end organ failure/severe impairment in function.

#### 2.3.4 Mini nutritional assessment (MNA)

The MNA categorizes patients scoring >24 points as having normal nutritional status, those with 17 to 23.9 points as being at risk for malnutrition, and those with fewer than 17 points as malnourished.^[35]^ The MNA test is composed of 4 measurements:

(1) Anthropometric measurements (weight, height, and weight loss);
(2) Global assessment (lifestyle, medication, and mobility);
(3) Dietary questionnaire (number of meals, food and fluid intake, and autonomy of feeding);
(4) Subjective assessment (self-perception of health and nutrition).

### 2.4 Sample size calculation

To investigate differences between study groups, sample size was estimated by GPOWER software. The resulting sample size, estimated according to a global effect size of 0.50 with type I error of 0.05 and a power of 80% was 128 patients.

#### 2.4.1 Statistical analysis

Categorical variables were described as frequencies and percentages (%), and continuous variables as mean with SD. A Chi-squared test and Student *t* test or the nonparametric Wilcoxon test was used to compare categorical and continuous variables, respectively. Hypothesis testing was 2-tailed. Analysis of variance with Scheffe test was used for analyze differences among different groups. Statistical significance was set at a level of *P* < .05. Pearson product-moment correlations were calculated to test associations among variables. Statistical analyses were performed using the SPSS statistical package (SPSS version 23.0 for windows). Multivariate regression analysis was performed to identify the independent effect of different variables on frailty status. In particular, the model was performed for evaluating the independent effect of age, gender, BMI, MoCA, GDS, FPG, BADL, IADL, NPI, CIRS-G comorbidity, and CIRS-G severity, MNA on frailty status.

## 3 Results

According to inclusion and exclusion criteria, a final population of 148 elderly patients, 75 cirrhotic CHILD A and 73 non-cirrhotic patients/controls, was suitable for the analysis. Of the total sample, 77 were males and 71 were females. Table 2 shows anthropometric and biochemical parameters of the study population. All participants were old (72.4 ± 5.7 years), slightly overweight (BMI = 26.7 ± 2.9 kg/m^2^) and had an education level mean of 7.4 ± 4.6 years. There was no significant difference in age, gender, systolic, and diastolic blood pressure, FPG, triglycerides and cholesterol levels between well-compensated liver cirrhotic and non-cirrhotic patients.

**Table 2:**
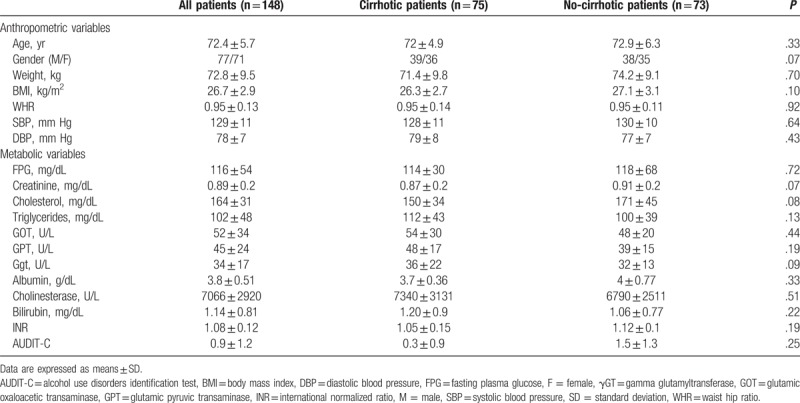
Anthropometric and biochemical parameters of the study participants.

Analyzing the cognitive performances in both study groups, we did not found significant alterations (Table 3). There are no patients affected by dementia and/or depression, as well as neither significant behavioral alteration was found at the NPI questionnaire. All patients also demonstrated an initial disability (activity daily living  = 5.2 ± 1.1 and IADL = 6.3 ± 1.1) without significant differences between the 2 groups (Table 3). We found no statistically significant differences between nutritional status in non-cirrhotic as compared with well-compensated liver cirrhotic group. Finally, CIRS-G scale score, comorbidity section, was similar between the 2 groups, showing moderate morbidity (2.6 ± 1.3), without significant differences between the 2 groups (Table 3). Conversely, CIRS-G scale score, severity section, was significantly different between 2 groups (Table 3).

**Table 3:**
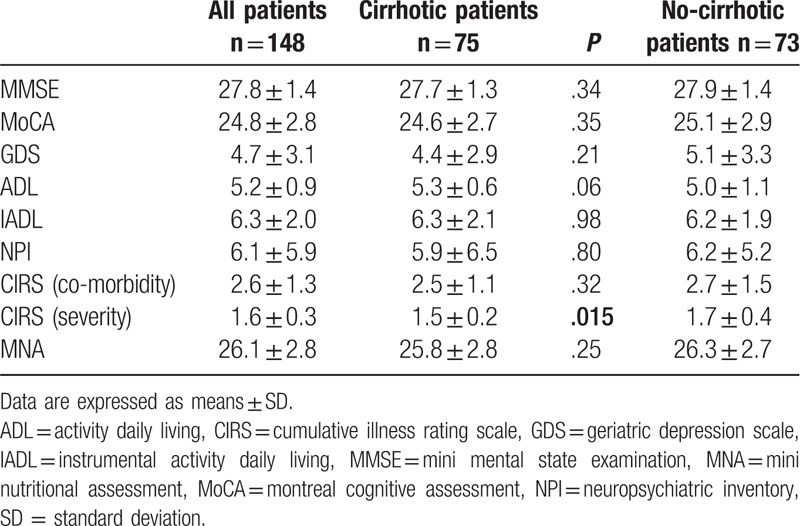
Cognitive assessment, comorbidities, and nutritional status assessment of the study participants.

Examining the Fried criteria for the frailty, 32 patients (21.6%) were classified as frail, 82 patients (55.4%) were classified as pre-frail and 34 patients (23.0%) were classified as no frail. No difference between the 2 groups regarding no frail (9.5% non-cirrhotic vs 13.5% cirrhotic; *P* < .167) and pre-frail (25.7% non-cirrhotic vs 29.7% cirrhotic; *P* < .107) diagnosis was observed. Conversely, frail diagnosis was significantly greater in non-cirrhotic group as compared with cirrhotic group (14.2% vs 7.5%; *P* < .05) (Table 4A).

**Table 4:**
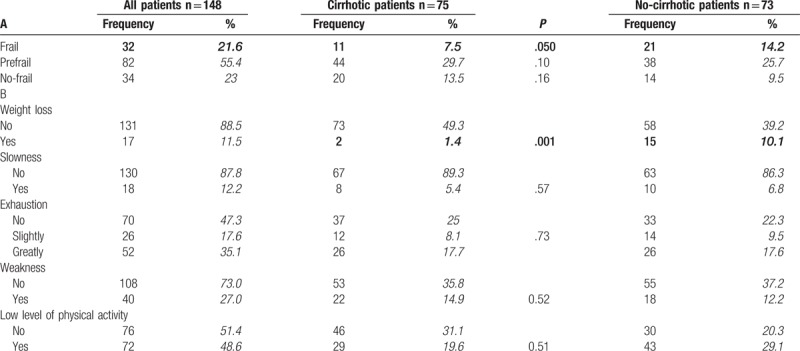
Prevalence of frailty criteria of the study participants.

Focusing on the specific components of the Fried Frailty score, 18 (12.2%) reported slowness, 52 (35.1%) exhaustion, 40 (27%) weakness, 72 (48.6%) low physical activity, without significant differences between the 2 groups. Only unintentional weight loss was statistically greater in non-cirrhotic group than cirrhotic group (10.1% vs 1.4%; *P* < .001) (Table 4B).

Furthermore, frailty status was significantly and positively associated with CIRS-G scale score, in both comorbidity and severity scores (*r* = 0.336, *P* < .001; *r* = 0.234, *P* < .004).

MNA showed a regular nutritional status in both groups (Table 3). Focusing on the specific components of MNA score, there was no significant difference in anthropometric measurements (7.8 ± 0.43 vs 7.9 ± 0.41; *P* < .073), in global assessment (7.9 ± 0.9 vs 7.6 ± 1.1; *P* < .153), in dietary questionnaire (7.5 ± 1.3 vs 7.3 ± 1.2; *P* < .995) respectively between non-cirrhotic group than well-compensated liver cirrhotic group. Conversely, well-compensated liver cirrhotic group showed a lower score of self-perception of health and nutrition as compared to non-cirrhotic group (3.2 ± 0.9 vs 2.7 ± 0.7; *P* < .002). Moreover, MNA showed nutritional status significantly and inversely associated with frailty (*r* = −0.482, *P* < .001), CIRS comorbidity (*r* = −0.289, *P* < 001), and CIRS severity scores (*r* = −0.242, *P* < 003).

Finally, the independent association of frailty status with anthropometric, cognitive, and nutritional parameters was evaluated by multivariate analysis. A model including age, gender, BMI, MoCA, GDS, BADL, IADL, NPI, CIRS-G comorbidity, and CIRS-G severity, MNA, as independent variables, explained 51% of frailty status variability. In such analysis, only female gender, BMI and MNA score were significantly and independently associated with frailty status (*β* = 0.177, *P* < .020; *β* = 0.231, *P* < .001; *β* = −0.315, *P* < .001) (Table 5).

**Table 5:**
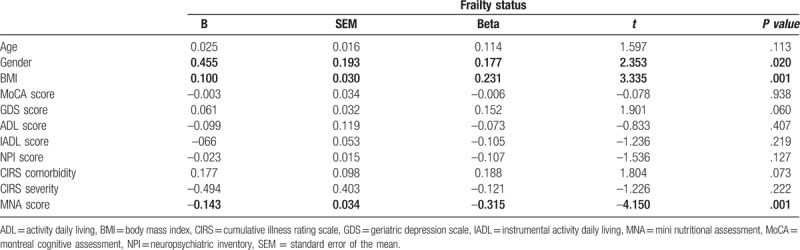
Linear multivariate analyses with frailty status as dependent variable.

## 4 Discussion

The present study investigated the frailty in non-cirrhotic elderly patients compared to well-compensated liver cirrhotic elderly patients, and demonstrated several important findings.

First, the major finding of this study is that, according to the Fried criteria, non-cirrhotic patients, unexpectedly, were frailer than well-compensated liver cirrhotic patients. Secondly, did not find statistically significant differences concerning the incidence of slowness, weakness, physical activity, and gait-speed among the 2 study populations. Nonetheless, it is interesting to note how both groups showed altered results as fatigue and low physical exercise. Third, the most represented Fried criterion was the unintentional weight loss in non-cirrhotic patients compared to well-compensated liver cirrhotic patients. Fourth, CIRS severity score was significantly and positively associated with frailty status, most likely due to the high prevalence of cardiovascular disorders in non-cirrhotic elderly patients. Fifth, the main determinants of frailty variability were female gender, BMI, and nutritional status.

To our knowledge, this is the first study that investigates the frailty in well-compensated liver cirrhotic elderly patients suggesting that liver disease, if not in an advanced state, should not raise particular concerns regarding the management of these patients, besides the normal medical controls required by pathologies from which they are affected.

Frailty has been well studied in the geriatric population as the most powerful predictor of decline and vulnerability of several physiologic systems and mostly of disability, hospitalization, and death.^[36]^ Although in most studies, in elderly cirrhotic patients, frailty assessment was used only to assess the prognosis of liver disease and to predict outcomes and mortality in the cirrhotic population,^[9–11]^ in clinical practice, patients with liver cirrhosis were always considered “frailer” when compared to elderly patients. This condition is due to a greater tendency to malnutrition, sarcopenia, weakness, metabolism, and abnormalities of the immune system, as well as an increased need for hospitalizations for complications of liver diseases such as encephalopathy, ascites or varicose vein bleeding.^[37]^ In most studies, moreover, frailty is not evaluated according to the Fried criteria.^[3]^ Despite the fact that European and US societies in geriatric medicine recommends that all elderly patients should be screened for frailty, unfortunately, there is no consensus as to how frailty should be assessed.^[38]^ It is common that when used the term “frailty,” frailty assessment was based on clinical evaluation, almost as an attempt to assess the patient's frailty based only on simple visual appearance. As well as other researchers, Tapper et al,^[7]^ evaluating how and whether the frailty has an impact after transplant, measure the frailty by using assessment tools of ability to complete activities of daily living, of risk of developing bedsores, and the likelihood of falling. At the same time, they not used Fried criteria.

Therefore, although currently focused on transplant candidates, the frailty assessment could be useful to other patients with cirrhosis, especially for well-compensated liver cirrhotic patients/CHILD-A. Considering our findings, it would be suggested to adopt and to incorporate into clinical practice, always and in the future, the use of frailty evaluation tests, according to Fried criteria, as in elderly non-cirrhotic patients.

Of all the frailty parameters evaluated, it was interesting to note that fatigue and low exercise were altered in both groups. Generally, fatigue is associated with worsening of health-related status, in close interaction among pain, depression, and chronic diseases. Instead, it is well known that elderly cirrhotic patients have a high incidence of fatigue, and, often, just this symptom is the most common extra-hepatic manifestation of HCV infection.^[39,40]^ Also in elderly non-cirrhotic patients, fatigue is a common symptom. It is likely that the concomitant muscle mass loss and the reduction of energetic substrates could explain the presence of fatigue and the limited exercise capacity that often characterizes subjects with and without liver disease.^[41]^ Conversely, in elderly patients, weight loss is usual and may be due to inadequate dietary intake, diseases, psychosocial factors, physiological free fat mass loss and sarcopenia.^[42,43]^ Likewise, cirrhotic patients have global malnutrition, muscle wasting and sarcopenia, present mostly in almost every patient with alcoholic cirrhosis but frequent in most other types of cirrhosis.^[44]^

Therefore, just for patients with cirrhosis, there are specific dietary guidelines because it is critically important for cirrhotic patients to maintain their muscle mass. Thus, paradoxically, to prevent muscle wasting, in the cirrhotic patients, the alimentary intake is more adequate just in protein intake.^[45–47]^ This is probably related to the fact that these subjects benefit from nutritional education/advice coming from the scheduled hepatology visits. In our study, MNA score shown that all patients were classified as not a risk for malnutrition or malnourished, with a better nutritional status in non-cirrhotic patients than in cirrhotic elderly but without significant differences. Moreover, nutritional status was also independently associated with medical comorbidity and severity. Importantly, there is increasing interest in nutritional interventions to improve poor nutrition and weight loss in elderly patients and in elderly frail patients. Identifying the elderly patients at risk of malnutrition is challenging due to the difficulty of nutritional assessment tools used, and the criteria to make a diagnosis of malnutrition. Nevertheless, the management of older people at risk of malnutrition should be multi-disciplinary and supported by appropriate nutritional advice and support.

Lastly, our study found that all patients were affected by initial disability, and by more comorbidity, without significant differences between the 2 groups. We also found that comorbidity and severity (CIRS-G score) were associated with frailty status, most likely due to the high prevalence of cardiovascular disorders in non-cirrhotic patients. Overall, if cirrhotic conditions confer protection against or accelerate coronary atherosclerosis has been an unresolved controversy, though it has been reported that the heart and the liver interact with each other. Shim et al^[48]^ demonstrated that the cardiovascular disorders among well-compensated liver cirrhotic patients did not differ significantly from non-cirrhotic patients. This could be explained by the favorable vascular profile in cirrhotic patients represented by hemostatic defects such as impaired coagulation, platelet dysfunction, and low blood pressure as well as by the lowest cholesterol levels.^[48]^ However, the present study design cannot unravel the potential reasons for such associations between comorbidity and frailty status. Speculatively, the effect of comorbidity on frailty may be due to direct effect on the body composition and probably on the adverse effect of concomitant medications. Although the association between comorbidity and frailty is intriguing, several points should be considered when interpreting these findings: we found no significant differences between frailty and the 14 items of the chronic medical illness (“morbidity”) that coexist in elderly non-cirrhotic patients compared to cirrhotic patients. The high prevalence of morbidity severity may warrant the interpretation of these finding. The present results indicate that frailty is associated with an elevated severity morbidity load, as assessed by CIRS score. Furthermore, a potential effect of the dietary intake on body weight and nutritional status should have been taken into account.

Indeed, despite the significant association between comorbidity and frailty status, a multivariate analysis, clearly, confirmed that nutritional status was independently associated to frailty status variability, thus suggesting that the potential impact of correct dietary intake in cirrhotic patients should be considered as a main determinant factor of non-frailty. In fact, elderly cirrhotic patients follow a diet that is certainly more adequate than that taken by the elderly non-cirrhotic patient.

Thus, based on the exposed data, we observed that paradoxically non-cirrhotic elderly patients are frailer than elderly well-compensated liver cirrhotic patients. In contrast, in everyday life, the elderly cirrhotic patient is considered frailer than non-cirrhotic elderly patient, but relying solely on “mere visual appearance.” Therefore, assessing frailty may be important for this particular patient category, to define a “clinical staging” and consequently to provide a most appropriate therapeutic indication. This clinical approach could; therefore, allow also the use of drugs not usually prescribed for the elderly cirrhotic patients. Only then, elderly cirrhotic patients will benefit from the best clinical and therapeutic practices that otherwise the physicians would not have practice out of fear of being too aggressive.

Finally, our study has some limitations that must be considered. First, this study includes a small sample size of the participants. Second, this is a cross sectional study showing only an association preventing to affirm a cause-effect relationship. However, the findings need to be confirmed in larger and longitudinal study.

## Author contributions

**Conceptualization:** Alessandro Federico, Maria Rosaria Rizzo.

**Data curation:** Alessandro Federico, Giuseppe Gerardo Caprio, Anna Maria Dalise, Marcello Dallio, Maria Rosaria Rizzo.

**Formal analysis:** Michelangela Barbieri.

**Investigation:** Alessandro Federico, Maria Rosaria Rizzo.

**Methodology:** Alessandro Federico, Giuseppe Gerardo Caprio, Anna Maria Dalise, Marcello Dallio, Maria Rosaria Rizzo.

**Project administration:** Maria Rosaria Rizzo.

**Software:** Michelangela Barbieri.

**Supervision:** Carmelina Loguercio, Giuseppe Paolisso, Maria Rosaria Rizzo.

**Validation:** Carmelina Loguercio, Giuseppe Paolisso.

**Writing – original draft:** Alessandro Federico, Maria Rosaria Rizzo.

**Writing – review and editing:** Maria Rosaria Rizzo.

Maria Rosaria Rizzo orcid: 0000-0002-1023-4260.
